# Emotional regulation ability and depressive symptoms among Chinese junior high school students: a cross-sectional analysis of the role of stress coping styles

**DOI:** 10.3389/fpsyt.2026.1917988

**Published:** 2026-10-14

**Authors:** Jingjing Wang, Liuhao Yang, Ruru Guo, Dongling Liu, Mengge Li

**Affiliations:** Department of Community Nursing, School of Nursing and Health, Zhengzhou University, Zhengzhou, Henan, China

**Keywords:** adolescent mental health, depressive symptoms, emotional regulation ability, junior high school students, stress coping ability

## Abstract

**Background:**

While emotional regulation and coping styles influence adolescent depression, the mediating role of coping styles in the link between emotional regulation ability and depressive symptoms remains underexplored among junior high school students-a critical developmental stage marked by immature coping patterns.

**Objectives:**

This study explores the correlations among emotional regulation ability, stress coping styles (problem-focused and emotion-focused coping), and depressive symptoms among junior high school students and examines the mediating effect of stress coping styles on the relationship between emotional regulation ability and depressive symptoms.

**Methods:**

A total of 600 junior high school students were recruited between September and December 2024 through a multi-school convenience sampling approach involving five junior high schools across three cities in Henan Province. A self-designed general information questionnaire, the Patient Health Questionnaire Depression Scale (PHQ-9), the Adolescent Coping Style Scale and the Emotional Regulation Ability Questionnaire were used in the investigation. Pearson correlation analysis was used to examine the associations among problem-focused coping, emotion-focused coping, emotional regulation ability, and depressive symptoms, and a cross-sectional indirect association analysis was conducted to examine whether the two coping styles statistically accounted for indirect associations between emotional regulation ability and depressive symptoms.

**Results:**

Among the 471 valid responses received, emotional regulation ability was significantly negatively associated with depressive symptoms (*r* = -0.523, *P* < 0.001). In the parallel mediation analysis, emotional regulation ability was significantly associated with problem-focused coping (B = -0.0733, *P* = 0.023) and was directly and negatively associated with depressive symptoms (B = -0.1283, *P* < 0.001), but was not significantly associated with emotion-focused coping (B = -0.0465, *P* = 0.067). The total indirect effect was not significant (effect = -0.0002, 95% CI: -0.0038-0.0033]), thus indicating that neither coping style significantly mediated this association.

**Conclusion:**

This study demonstrated that emotional regulation ability is significantly and negatively associated with depressive symptoms among junior high school students. However, neither problem-focused coping nor emotion-focused coping significantly mediate the relationship between emotional regulation ability and depressive symptoms. These findings indicate that greater emotional regulation ability is associated with fewer depressive symptoms in early adolescence, whereas stress coping styles may not function as stable mediators at this developmental stage. These results support prioritizing the evaluation of developmentally appropriate emotional regulation programs for junior high school students in school and community settings.

## 1 Introduction

Adolescence is a critical developmental period that is characterized by profound physical, psychological, and social changes. This period typically extends from puberty to early adulthood. For junior high school students, especially those in mid-adolescence, this stage involves simultaneous pressures resulting from rapid physiological maturation and increasing psychological autonomy. The emotional management and stress-coping abilities of such students are still developing, thus increasing their vulnerability to both external stressors and internal mood fluctuations; in turn, these factors can challenge effective emotional management during this period (1).

In China, junior high school students face a uniquely competitive academic environment marked by heavy workloads, frequent exams, and high parental and societal expectations. This chronic academic pressure significantly increases emotional distress and risk of depression. In addition to academic and interpersonal stressors, the psychological consequences of the COVID-19 pandemic may represent another important contextual factor affecting adolescent mental health. Previous studies have reported that adolescents experienced persistent emotional distress, including increased depressive symptoms and difficulties in psychological adjustment, after the COVID-19 pandemic. Therefore, considering post-pandemic psychological influences may provide a broader context for understanding depressive symptoms among adolescents (2, 3).

Compared with adolescents in other developmental stages or cultural contexts, Chinese junior high school students are especially vulnerable to stress-related emotional problems, due to the convergence of academic demands and ongoing physical and psychological development. This distinctive context highlights the importance of identifying protective factors, such as emotion regulation ability and adaptive coping styles in this population.

Depressive symptoms represent one of the most common psychological concerns faced by adolescents and commonly result in a persistent low mood that is accompanied by somatic features such as reduced appetite and fatigue (4). Negative affective experiences, including loneliness, anxiety, feelings of inferiority, shyness, and jealousy are also widespread within this population and serve as key clinical indicators of depression (5). Indeed, depressive symptoms represent a major modifiable risk factor for adolescents’ physical and mental health in the 21st century. In the Chinese context, epidemiological studies have reported prevalence rates among Chinese adolescents ranging from 19.85% to 22.2% (6, 7), and national trends have indicated both a steady annual increase and an increasingly early age of onset. Without timely, evidence-informed intervention, depressive symptoms among Chinese junior high school students may exacerbate the emotional distress faced by such students and progress to clinical conditions such as major depressive disorder or generalized anxiety disorder. Untreated symptoms are further linked to adverse outcomes, including academic disengagement, social withdrawal, substance misuse, and nonsuicidal self-injury (8, 9). Notably, research among Chinese adolescents has confirmed that improving stress coping ability positively reduces depressive symptoms among adolescents (10, 11).

The term stress coping style refers to the relatively stable cognitive and behavioral patterns that individuals employ to manage external stressors (e.g., life changes, academic pressure, and interpersonal conflicts) and the accompanying negative emotional distress (12). Rooted in the transactional model of stress and coping, coping represents a dynamic psychological process that integrates cognitive appraisal, situational evaluation, and adaptive behavioral responses, with the core goals of reducing stress impact, restoring emotional equilibrium, and maintaining psychological homeostasis (13). A widely recognized classification distinguishes problem-focused coping, which targets the root causes of stress to resolve the situation directly, and emotion-focused coping, which aims to regulate distressing emotions rather than change the stressor itself (13). Extensive evidence indicates that daily stress is positively associated with depressive symptoms among middle school students (14). Adaptive coping strategies, including problem-solving and supportive emotion-focused approaches, can buffer the detrimental effects of stress on depression. In contrast, maladaptive coping strategies such as avoidance, emotional outbursts, and passive discharge are linked to elevated depressive levels under persistent stress (15).

Previous studies have confirmed that positive coping strategies can effectively alleviate depressive symptoms, whereas negative strategies not only fail to help but can also exacerbate such symptoms. Xie et al. surveyed 4,548 Chinese high school students and revealed a bidirectional negative association between positive coping and depressive symptoms: faster decreases in positive coping predict greater increases in depressive symptoms. In contrast, maladaptive coping unidirectionally predicts depressive symptoms, namely, slower declines in maladaptive coping are linked to more severe symptom increases (16). Longitudinal analyses of national adolescent health data have further revealed that emotion-focused coping mediates the effect of life stress on depressive symptoms. Specifically, adaptive emotional coping (e.g., seeking social support and expressing emotions) buffers stress-related depression, whereas maladaptive emotional coping (e.g., avoidance, self-blame, and emotional outbursts) strengthens the stress–depression link (17). Thus, coping style is strongly associated with depressive symptoms, and under certain conditions, emotion-oriented coping serves as a key mediator in this relationship. However, the precise pathways through which coping styles influence depressive symptoms remain unclear.

Two core theoretical frameworks underpin this study: the transactional model of stress and coping, and emotional regulation theory. Proposed by Lazarus and Folkman (13) in 1984, the transactional model views stress not as a direct effect of external stressors, but as a dynamic interaction between individuals and their environment. It defines coping as cognitive and behavioral efforts to manage stressors and associated distress, and positions it as central to mediating the link between stress and mental health outcomes. Over the past four decades, this model has become foundational for understanding stress responses and how coping strategies shape psychological well-being, particularly among adolescents (14, 15). Emotional regulation theory, first articulated by Gross (18) in the 1990s, defines emotional regulation as the conscious or unconscious modulation of the generation, intensity, duration, and expression of emotional responses. It identifies effective emotional regulation as a key protective factor against psychological distress, supporting adaptation to situational demands and emotional balance. Research grounded in this theory consistently links stronger emotional regulation ability with lower depressive and anxiety symptoms in adolescents (19). Together, these theories provide the conceptual foundation for examining the relationships among emotional regulation ability, coping styles, and depressive symptoms in this study.

Beyond the role of stress coping styles in adolescent depressive symptoms, emotional regulation ability represents another core self-regulatory construct that is closely intertwined with both coping processes and mental health outcomes. Emotional regulation ability refers to an individual’s inherent and trainable capacity to consciously modulate the generation, intensity, duration, and expression of their own emotional responses through cognitive, behavioral, or physiological strategies (18). This foundational psychological process focuses on maintaining emotional balance, preventing emotional dysregulation (e.g., excessive anger or prolonged sadness), and adapting emotional reactions to situational demands without relying on external interactions or goal achievement. Core functions of this ability include alleviating negative emotions (e.g., anxiety and frustration) and sustaining adaptive positive emotions (e.g., calmness and resilience) with the aim of supporting psychological well-being and daily functioning.

Both stress coping style and emotional regulation ability are rooted in the self-regulation system and aim to reduce stress-induced negative emotions and maintain psychological well-being. Individuals first appraise a stressor’s nature and controllability and then select an appropriate coping strategy, in which context they actively engage in emotional regulation to manage emerging emotions (19). Coping style involves external behavioral strategies, whereas emotional regulation involves the internal processing of stress-related emotions (20) Emotional regulation is closely related to the effectiveness and adaptability of coping strategies; thus, it may contribute to stress-related outcomes, such as depressive symptoms, through its association with coping style selection (21).

Existing studies have begun to disentangle the links among emotional regulation, coping styles, and depressive symptoms in adolescents, yet inconsistent findings and limited developmental evidence remain. A study of 142 high school students identified emotional regulation ability as the strongest predictor of depressive symptoms; however, coping style was not observed mediate this relationship (22). Another study of 312 high school students revealed that coping strategies, such as seeking support, problem solving, and avoidance mediate the relationship between perceived stress and positive emotions and that emotion regulation plays a mediating role in this context (23). Importantly, however, these prior studies are largely limited to high school samples, and few have directly tested whether coping styles mediate the association between emotional regulation ability and depressive symptoms specifically among junior high school students. Accordingly, this critical gap leaves unclear whether findings from older adolescents can be generalized to early adolescence, and the present study was designed to directly address this limitation by examining the mediating role of stress coping styles in a junior high school population.

Taken together, existing research on emotional regulation ability, stress coping styles, and depressive symptoms has critical limitations. Although prior studies link coping styles to adolescent depression, and identify emotion-oriented coping as a mediator between stress and depression the mechanism by which coping styles connect emotional regulation ability to depressive symptoms remains unclear. Moreover, most studies focus on high school students; it is unknown whether the established mediation model applies to junior high school students, who are at an earlier, distinct developmental stage. We hypothesized that emotional regulation ability would be negatively associated with depressive symptoms among junior high school students, and that both problem-focused and emotion-focused coping would independently mediate this relationship. Accordingly, this cross-sectional study examined the association between emotional regulation ability and depressive symptoms among Chinese junior high school students and evaluated whether problem-focused and emotion-focused coping showed significant indirect associations within this cross-sectional model.

## 2 Methods

### 2.1 Study design and participants

This study used a multi-school cross-sectional design involving five junior high schools located in three cities in Henan Province. From September to December 2024, two schools were selected through a convenience sampling approach from a city in eastern Henan, two were selected from a city in central Henan, and one was selected from a city in southern Henan. All the classes in each school were numbered, and six classes were randomly selected to participate in the questionnaire survey. Students were therefore recruited within naturally occurring class clusters, with students nested within classes and classes nested within schools.

Before the assessment, the students in these classes returned a parent questionnaire and an instruction manual to inform their parents of the study and obtain their consent. Students participated in this research voluntarily, and their legal guardians provided written informed consent; at that point, the students completed academic tests and paper-and-pencil questionnaires during their regular class time under the guidance and supervision of two trained researchers. In addition, one classroom teacher helped administer the survey. This procedure complied with the ethics guidelines of the author’s institutional ethics committee. A total of 600 questionnaires were distributed; 574 questionnaires were returned, and those that contained logical contradictions or duplicate responses were excluded. Ultimately, 471 valid questionnaires remained, for an effective response rate of 82.06%.

The inclusion criteria were as follows: (1) students who had been admitted to junior high school; (2) those who had the ability to read, comprehend and complete the questionnaire independently; and (3) those who had provided a written informed consent form that had been signed by their legal guardians and agreed to participate voluntarily in this research.

The exclusion criteria were as follows: (1) individuals who failed to complete the research investigation throughout the process because of suspended studies, school transfers, absences, and similar factors during the survey; and (2) those who were unable to understand and complete the questionnaire because of mental illness, cognitive impairment, physical illness, or similar issues.

### 2.2 Sample size determination

This research featured a quantitative, cross-sectional study design. The STROBE guidelines were followed to report this study (24).

The sample size calculation was based on the primary multiple regression model and the anticipated invalid questionnaire rate. Classes served as the sampling units during participant recruitment. The minimum sample size was estimated using G*Power 3.1 for a multiple regression model. The full outcome model included 17 predictors: emotional regulation ability, problem-focused coping, emotion-focused coping, and 14 demographic and behavioral covariates. With α = 0.05, statistical power (1-β) = 0.90, and an expected medium effect size of Cohen’s f² = 0.15, the minimum required sample size was 179 participants. The final analytic sample of 471 participants therefore exceeded the minimum requirement. This calculation was intended for the primary regression model and was not a dedicated power analysis for detecting small indirect effects or for accounting for the loss of precision associated with clustered sampling.

As a supplementary check, the commonly used rule of thumb of 10–20 participants per study variable was also considered. With 19 variables included in the study, this criterion suggested a sample size of 190–380 participants. After allowing for an anticipated 15% invalid questionnaire rate, the target recruitment size was increased accordingly. This study featured a total of 19 variables, including 15 general information items, 1 depressive symptoms dimension, 2 coping-style dimensions, and 1 emotional regulation ability dimension. Therefore, the required sample size was estimated to be 190–380 participants. Given the assumption of a 15% invalid questionnaire rate, the target sample size was adjusted to 224-448. In total, 600 questionnaires were distributed, and 471 valid questionnaires were included in the final analysis, thus indicating that the sample size was adequate for the planned analyses.

### 2.3 Questionnaire

The initial questionnaire was developed based on a systematic literature review and a pilot survey conducted among 40 junior high school students who were recruited from a school not included in the formal study sample, with no overlap between pilot participants and the main study sample. The pilot survey was conducted to evaluate the clarity, appropriateness, and comprehensibility of questionnaire items. Based on the pilot results and group discussions among the research team, necessary revisions were made to optimize item expression, adjust item order, and improve the overall readability and applicability of the questionnaire for junior high school students. The final questionnaire includes four main modules:

1. Demographic characteristics: gender, grade, father’s educational level, mother’s educational level, family residence location (rural/urban), only-child status, living arrangement, academic ranking, student leader status, number of close friends, weekly electronic device use, daily sleep duration, and physical activity frequency. These demographic variables were selected based on prior empirical evidence indicating their associations with emotional regulation, stress coping, and depressive symptoms in adolescents (25).
2. Patient Health Questionnaire-9 (PHQ-9): This questionnaire was developed by Kroenke et al. (26) in 2001. It features 9 items, each of which is scored on a scale ranging from 0 to 3; total scores range from 0 to 27, and higher scores indicate more severe depression. Scores ≥10 suggest the presence of clinically significant depressive symptoms with high sensitivity and specificity. This scale has been validated for use among Chinese junior high school students by Kong et al. (25), and it exhibits strong reliability and validity with respect to this population. The Cronbach’s α coefficient for this tool was 0.911 in this study.
3. This study selected the Middle School Students’ Coping Style Scale developed by Chen et al. (2000) because it is the most widely used and well-validated tool for assessing stress coping styles among Chinese adolescent populations (27). This measure was developed specifically for middle school students and captures both adaptive and maladaptive coping patterns, which aligns with the theoretical framework of the transactional model of stress and coping and supports the study’s aim to classify problem-focused and emotion-focused coping styles. It includes 36 items that are scored on a 4-point Likert scale ranging from 1 (“not adopted”) to 4 (“often adopted”). The scale includes seven dimensions that form two coping subscales. Problem-focused coping comprises problem-solving (7 items), seeking support (7 items), and reasonable explanation (5 items), for a total of 19 items. Emotion-focused coping comprises tolerance (4 items), avoidance (4 items), emotional release (4 items), and fantasy/denial (5 items), for a total of 17 items. The scores of the corresponding dimensions were summed to obtain the problem-focused coping and emotion-focused coping scores used in the present analyses. The Cronbach’s α coefficients for each dimension and subscale included in this scale are as follows: 0.83 for problem-solving, 0.78 for seeking support, 0.78 for reasonable explanation, 0.67 for tolerance, 0.68 for avoidance, 0.74 for emotional release, 0.76 for fantasy/denial, and 0.92 for the overall scale. The split-half reliability coefficient is 0.88. After an 8-week interval, the retest correlation coefficient between the two tests was 0.89. In the present study, the Cronbach’s α coefficients were 0.919 for the 19-item problem-focused coping subscale and 0.869 for the 17-item emotion-focused coping subscale, indicating good internal consistency for both variables.
4. Junior High School Students’ Emotional Regulation Ability Scale: This measure was developed by Zhang et al. (28) in 2016. It consists of 5 dimensions: seeking external support, positive remediation, controlling negative suggestions, rational emotional regulation, and controlling negative venting. The items are scored on a 5-point Likert scale ranging from 1 (“strongly disagree”) to 5 (“strongly agree”) across a total of 17 questions. The higher the scores on this questionnaire are, the better junior high school students’ emotional regulation ability is. The homogeneity reliability of the questionnaire is 0.836, the split-half reliability is 0.674, and the internal consistency coefficients of each dimension are 0.694, 0.769, 0.568, 0.781, and 0.692. The Cronbach’s α coefficient for this tool was 0.856 in this study.

### 2.4 Quality control

This study relied on a face-to-face questionnaire collection method. All the researchers completed a standardized, uniform training program that covered study purpose, questionnaire structure, item explanations, administration procedures, ethical requirements, and strategies for addressing student inquiries during completion; they then passed a standardized proficiency assessment to confirm consistent and accurate survey implementation. Under the guidance of trained researchers (who were assisted by junior high school teachers), unified completion instructions were provided, and real-time question-answering services were offered to help avoid errors based on an incomplete understanding. The questionnaires were distributed and collected onsite, and immediate preliminary checks were conducted to ensure that any correctable incomplete or ambiguous responses were marked and corrected. Valid questionnaires were coded, and a dual independent data entry method was adopted to reduce input errors. After the data were entered, they were strictly verified through logical checks and cross-validation, and invalid questionnaires (e.g., those that contained repeated responses, lacked key data, or were logically inconsistent) were excluded; finally, reliability and validity tests were conducted by reference to the final dataset.

Because the questionnaires were completed anonymously and contained no personally identifying information, individual PHQ-9 scores, including responses to item 9 concerning thoughts of death or self-harm, could not be retrospectively linked to specific students. To balance participant anonymity with the duty of care, trained researchers and school staff remained available during questionnaire administration, and all participants were informed of available psychological support resources and school-based support channels. Students who experienced psychological distress were encouraged to seek support from the school psychological support team. This procedure was implemented in accordance with the approved ethical protocol while protecting participants’ privacy and confidentiality.

### 2.5 Statistical analysis

Missing data were excluded from the final analysis. All the statistical analyses were conducted with the assistance of SPSS 26.0 software and the PROCESS macro for SPSS (Version 4.2 beta). Descriptive statistics were used to summarize the participants’ demographic characteristics. Continuous variables are presented in terms of means and standard deviations (*M* ± *SD*), and categorical variables are presented in terms of frequencies and percentages. Pearson’s correlation analyses were performed to examine the associations among depressive symptoms, emotional regulation ability, problem-focused coping, and emotion-focused coping. All tests were two-tailed, and *P* < 0.05 was considered to indicate statistical significance. Regression assumptions were assessed using residual diagnostics, variance inflation factors (VIFs), tolerance values, Durbin-Watson statistics, and Cook’s distance.

A cross-sectional indirect association analysis was conducted using PROCESS Model 4 to examine whether problem-focused coping and emotion-focused coping statistically accounted for indirect associations between emotional regulation ability and depressive symptoms. Emotional regulation ability was entered as the independent variable, depressive symptoms as the dependent variable, and problem-focused coping and emotion-focused coping as parallel mediators. The analysis controlled for the following covariates: age, gender, grade, only-child status, living arrangement, father’s educational attainment, mother’s educational attainment, place of origin, academic ranking, student leader status, number of close friends, time spent using electronic devices per week, sleep duration per day, and frequency of physical activity. Covariates were coded according to the questionnaire categories. Age was treated as a continuous variable. Gender, only-child status, place of origin, and student leader status were coded as binary variables. Grade was coded as 1-3; living arrangement as 1-3; paternal and maternal educational attainment as 1-6; academic ranking, number of close friends, weekly electronic device use, daily sleep duration, and physical activity frequency as 1-4. Unstandardized regression coefficients (B), standard errors (*SE*), *t* values, *p* values, and model statistics are reported. The indirect effects were tested using 95% confidence intervals on the basis of 5,000 bootstrapped samples. An indirect effect was considered to be significant if the 95% confidence interval did not include zero. Because class-level identifiers were not retained in the final analytic dataset, clustering could not be incorporated into the statistical models; therefore, within-class dependence may have affected the precision of standard errors and significance tests.

## 3 Results

To examine the primary aim of exploring the relationship among emotional regulation ability, stress coping styles, and depressive symptoms in Chinese junior high school students, we first conducted descriptive analyses of demographic and key variable characteristics.

### 3.1 Demographic characteristics

A total of 471 students were included in this research; their mean age was 13.52 ± 1.33 years. A total of 52.9% (n = 249) of the participants were female, and 46.1% (n = 217) were in Grade 7. The most common level of parental education was junior middle school for both fathers (49.7%, n = 234) and mothers (48.8%, n = 230), and 72.0% (n = 339) of the participants were from rural areas. The most common academic category was in the top 30% (36.9%, n = 174), and 40.6% (n = 191) of the participants reported serving as student leaders. Most students reported having 1–3 close friends (58.4%, n = 275), spending 2–4 h/week using electronic devices (33.3%, n = 157), sleeping 7–8 h/day (35.7%, n = 168), and engaging in physical activity daily (51.2%, n = 241). The most frequently reported unhealthy dietary habit was disliking fruit (91.9%, n = 433) (**Table 1**). Using the prespecified PHQ-9 cutoff score of ≥ 10, 387 of the 471 participants (82.2%) exceeded the screening threshold for elevated depressive symptoms.

**Table 1:** Characteristics of participants.

Variable	*N* (%)
Age, M (SD)	13.52 (1.33)
Gender	
Male	222 (47.1)
Female	249 (52.9)
Grade	
Grade 7	217 (46.1)
Grade 8	102 (21.7)
Grade 9	152 (32.3)
Only child	
Yes	44 (9.3)
No	427 (90.7)
Living arrangement	
Only parents	221 (46.9)
Only grandparents	70 (14.9)
Parents and grandparents	180 (38.2)
Father’s educational attainment	
Primary school or below	38 (8.1)
Junior middle school	234 (49.7)
Senior high school/secondary vocational/technical school	156 (33.1)
Junior college	26 (5.5)
Bachelor’s degree	15 (3.2)
Master’s degree or above	2 (0.4)
Mother’s educational attainment	
Primary school or below	69 (14.6)
Junior middle school	230 (48.8)
Senior high school/secondary vocational/technical school	124 (26.3)
Junior college	26 (5.5)
achelor’s degree	19 (4.0)
Master’s degree or above	3 (0.6)
Place of origin	
Rural	339 (72.0)
Urban/town	132 (28.0)
Academic ranking	
Top 5%	54 (11.5)
Top 30%	174 (36.9)
Top 70%	163 (34.6)
Others	80 (17.0)
Student leader	
Yes	191 (40.6)
No	280 (59.4)
Number of close friends	
0	41 (8.7)
1-3	275 (58.4)
4-5	80 (17.0)
> 5	75 (15.9)
Time spent using electronic devices (per week)	
0–2 h	118 (25.1)
2–4 h	157 (33.3)
4–6 h	92 (19.5)
≥ 6 h	104 (22.1)
Sleep duration (per day)	
≤ 6 h	63 (13.4)
6–7 h	140 (29.7)
7–8 h	168 (35.7)
≥ 8 h	100 (21.2)
Physical activity frequency	
Daily	241 (51.2)
Once every 2–3 days	134 (28.5)
Once per week	61 (13.0)
Rarely/never	35 (7.4)
Unhealthy dietary habits (multiple response)	
Prefers sugar-sweetened beverages	220 (46.7)
Prefers fried foods	265 (56.3)
Dislikes fruit	433 (91.9)
Dislikes vegetables	356 (75.6)
Skips breakfast	353 (74.9)
Picky eating	313 (66.5)

### 3.2 Correlation analysis among depressive symptoms, emotional regulation ability, problem-focused coping, and emotion-focused coping

To examine the bivariate relationships among emotional regulation ability, stress coping styles, and depressive symptoms and to provide a preliminary foundation for the subsequent mediation analysis, Pearson correlation analyses were conducted. Pearson correlation analyses revealed that emotional regulation ability was significantly negatively associated with depressive symptoms (*r* = -0.523, *p* < 0.001). Depressive symptoms were not significantly associated with problem-focused coping (*r* = 0.016, *p* = 0.732) or emotion-focused coping (*r* = 0.070, *p* = 0.131). In addition, emotional regulation ability was not significantly associated with problem-focused coping (*r* = -0.078, *p* = 0.091), but it was weakly and negatively associated with emotion-focused coping (*r* = -0.095, *p* = 0.039). Problem-focused coping was significantly positively associated with emotion-focused coping (*r* = 0.264, *p* < 0.001; see **Table 2**). These correlation results indicate that emotional regulation ability is closely linked to lower depressive symptoms, whereas the associations of coping styles with the key variables are weak or nonsignificant, providing initial support for the primary role of emotional regulation in this study model.

**Table 2:** Means, standard deviations, and Pearson correlations.

Variables	Mean	SD	1	2	3	4
Depression	15.11	5.69	—			
Emotional regulation ability	121.35	19.30	-0.523^***^	—		
Problem-focused coping	45.94	12.16	0.016	-0.078	—	
Emotion-focused coping	34.72	9.69	0.070	-0.095^*^	0.264^***^	—

SD, standard deviation; correlation coefficients are Pearson’s r. *p < 0.05, ***p < 0.001 (two-tailed).

### 3.3 Cross-sectional indirect associations through stress coping styles

A cross-sectional indirect association analysis using PROCESS Model 4 was conducted to examine whether significant indirect associations through problem-focused coping or emotion-focused coping were present after controlling for relevant covariates. Model diagnostics indicated no serious multicollinearity or influential observations, with VIFs ranging from 1.08 to 4.52, tolerance values from 0.221 to 0.929, Durbin-Watson statistics from 1.91 to 1.95, and a maximum Cook’s distance of 0.089. Emotional regulation ability was significantly and negatively associated with problem-focused coping (B = -0.0733, SE = 0.0321, t = -2.28, *p* = 0.023) but was not significantly associated with emotion-focused coping (B = -0.0465, SE = 0.0253, t = -1.84, *p* = 0.067) (**Table 3**). When emotional regulation ability, the two coping dimensions, and all covariates were simultaneously included in the regression model with depressive symptoms as the outcome, emotional regulation ability remained significantly and negatively associated with depressive symptoms (B = -0.1283, SE = 0.0123, t = -10.41, *p* < 0.001), whereas neither problem-focused coping (B = -0.0090, SE = 0.0185, t = -0.49, *p* = 0.625) nor emotion-focused coping (B = 0.0177, SE = 0.0234, t = 0.76, *p* = 0.450) was significantly associated with depressive symptoms. Bootstrap analyses on the basis of 5,000 resamples indicated that the total indirect effect was not significant (effect = -0.0002, BootSE = 0.0018, 95% *CI* [-0.0038, 0.0033]) (**Table 4**). The specific indirect effects via problem-focused coping (effect = 0.0007, BootSE = 0.0014, 95% *CI* [-0.0021, 0.0038]) and emotion-focused coping (effect = -0.0008, BootSE = 0.0012, 95% *CI* [-0.0037, 0.0013]) were also not significant. These findings suggest that neither problem-focused coping nor emotion-focused coping significantly mediated the relationship between emotional regulation ability and depressive symptoms (**Figure 1**).

**Table 3:** Regression models for the cross-sectional indirect association analysis (PROCESS Model 4).

Variables	Step 1: Problem-focused coping		Step 2: Emotion-focused coping		Step 3: Depressive symptoms	
	B	*t*	B	*t*	B	*t*
Age	-0.0399	-0.034	-0.1888	-0.202	-0.9930^*^	-2.199
Gender	-0.9280	-1.066	0.0931	0.136	-0.1783	-0.538
Grade	0.3341	0.245	-1.4956	-1.394	0.8566	1.649
Only child	-0.3943	-0.194	-1.8508	-1.153	1.3286	1.711
Living arrangement	0.0911	0.142	0.6139	1.216	0.0107	0.044
Father’s educational attainment	-0.6249	-0.719	0.3512	0.513	-0.6269	-1.894
Mother’s educational attainment	-0.1558	-0.199	-0.0108	-0.018	0.2878	0.967
Place of origin	0.1355	0.091	1.7740	1.520	0.8212	1.453
Academic ranking	-0.6700	-0.979	0.2624	0.487	0.2189	0.839
Student leader	-0.0056	-0.005	-0.9718	-0.999	-0.8881	-1.888
Number of close friends	0.3663	0.534	0.3636	0.674	-0.5247^*^	-2.011
Time spent using electronic devices (per week)	-1.0160	-1.873	-0.2772	-0.649	0.2418	1.167
Sleep duration (per day)	-0.2776	-0.413	-1.4509^**^	-2.742	-0.5654^*^	-2.194
Physical activity frequency	-0.4388	-0.709	-0.5281	-1.084	0.7990^***^	3.390
Emotional regulation ability	-0.0733^*^	-2.280	-0.0465	-1.839	-0.1283^***^	-10.413
Problem-focused coping	—	—	—	—	-0.0090	-0.490
Emotion-focused coping	—	—	—	—	0.0177	0.757
R²	0.0266		0.0494		0.3591	
F	0.8292		1.5756		14.9306^***^	

B, unstandardized regression coefficient. *p < 0.05, **p < 0.01, ***p < 0.001.

**Table 4:** Total, direct, and indirect effects of emotional regulation ability on depressive symptoms through problem-focused coping and emotion-focused coping (bootstrap estimates).

Effects	Estimate	SE/BootSE	95% *CI*
Total indirect effect	-0.0002	0.0017	[-0.0038, 0.0034]
Direct effect (c′)	-0.1283	0.0123	[-0.1525, -0.1041]
Indirect effect via problem-focused coping	0.0007	0.0014	[-0.0021, 0.0038]
Indirect effect via emotion-focused coping	-0.0008	0.0012	[-0.0036, 0.0014]

Bootstrap 95% confidence intervals were generated using 5,000 bootstrap resamples. For indirect effects, mediation is significant when the 95% confidence interval does not include zero.

**Figure 1:**
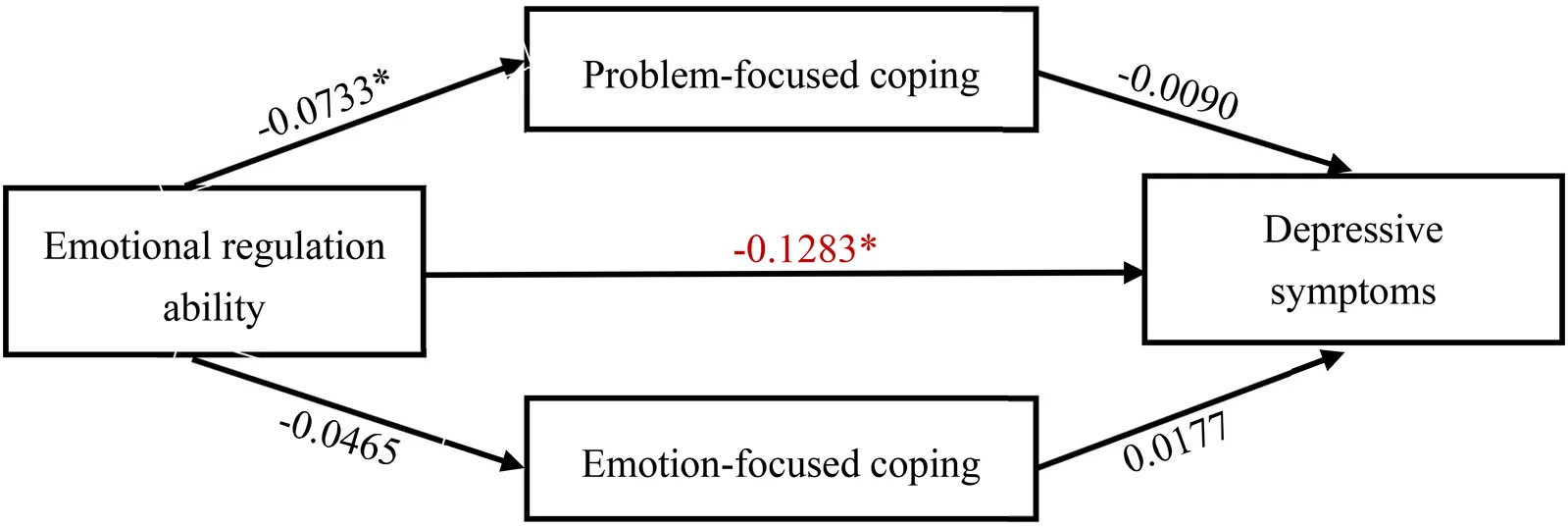
Cross-sectional indirect association model of emotional regulation ability, problem-focused coping, emotion-focused coping, and depressive symptoms. *p < 0.05, ***p < 0.001. Path values are unstandardized regression coefficients. The model controlled for age, gender, grade, only-child status, living arrangement, parental education, place of origin, academic ranking, student leader status, close friends, electronic device use, sleep duration, and physical activity.

Taken together, these results show that emotional regulation ability is independently and negatively associated with depressive symptoms among junior high school students, while no significant indirect association through either coping style was observed in the cross-sectional model. This key finding provides clear empirical evidence to advance understanding of the links between emotional regulation, coping, and depressive symptoms in early adolescence.

## 4 Discussion

This multi-school cross-sectional study examined associations among emotional regulation ability, stress coping styles, and depressive symptoms in Chinese junior high school students, testing whether problem-focused or emotion-focused coping mediates the link between emotional regulation and depression. As hypothesized, emotional regulation ability was significantly and negatively associated with depressive symptoms. Contrary to our hypothesis, however, neither coping style significantly mediated this relationship. These findings clarify the negative association between emotional regulation ability and depressive symptoms and the lack of a significant mediating role for coping styles in early adolescence.

The results of the present study revealed that emotional regulation ability is significantly and negatively associated with depressive symptoms among junior high school students, thus indicating that students with greater emotional regulation ability experience fewer depressive symptoms. These results are consistent with previous studies reporting that greater emotional regulation ability is associated with better mental health outcomes among adolescents (29). According to self-regulation system theory, emotional regulation ability, as a core component of individual self-regulation, can help individuals effectively identify, suppress and adjust to negative emotions, thereby reducing the occurrence of depressive symptoms. In the case of junior high school students, who are undergoing a special period of physical and mental development, their emotional systems are not yet mature, and they are susceptible to emotional imbalance and negative emotions accumulation when they encounter academic pressure and interpersonal troubles (30). Greater emotional regulation ability has been associated with fewer depressive symptoms among adolescents experiencing stress (31, 32). Our results further support a significant negative association between emotional regulation ability and depressive symptoms among junior high school students, highlighting the relevance of emotional regulation to mental health during early adolescence.

Contrary to our hypothesis, neither problem-focused coping nor emotion-focused coping play a significant mediating role in the relationship between emotional regulation ability and depressive symptoms. These results are inconsistent with those that have been reported by certain studies of both senior high school students and adults (11, 16) but are consistent with the findings of researchers who have reported that coping styles may not mediate the relationship between emotional regulation and depression in early adolescence (22). Several factors may explain these results. First, junior high school students are in the stage of early adolescence, and their stress coping styles are still developing, which may reduce the stability of coping patterns and weaken their indirect associations with depressive symptoms. Second, the indirect effects observed in mediation models are often smaller than direct effects, and our sample size calculation was based on the primary regression model rather than a dedicated analysis of indirect effects. Therefore, the statistical power to detect small mediation effects may have been limited. Third, the cross-sectional design cannot establish temporal relationships among emotional regulation ability, coping styles, and depressive symptoms (16, 21). Third, this study features a cross-sectional design and cannot clarify the temporal sequence among the variables considered in this context; however, the mediating effect is a dynamic process that requires longitudinal data for verification (33). Fourth, in light of the unique environment faced by Chinese junior high school students, which is characterized by academic pressure, the associations of coping styles with depressive symptoms may be weaker, whereas emotional regulation ability may show a more direct association with depressive symptoms in this context (34). In addition, the correlation analysis conducted for this study revealed that the correlation between coping styles and depressive symptoms is weak, thus providing an explanation for the nonsignificant mediating effect observed in this context.

Although emotional regulation ability was significantly associated with problem-focused coping, no significant indirect association with depressive symptoms through either coping style was observed. The unexpected negative association between emotional regulation ability and problem-focused coping requires further interpretation. Although problem-focused coping is generally considered an adaptive strategy, emotional regulation ability and coping style represent distinct self-regulatory processes. Among junior high school students, individuals with higher emotional regulation ability may rely less on explicit problem-solving strategies because they may regulate emotional responses internally before adopting external coping behaviors. The small effect size and cross-sectional design limit the interpretation of this association, and future longitudinal studies are needed to clarify the developmental relationship between these constructs.

These findings suggest that problem-focused coping alone may not explain the association between emotional regulation ability and depressive symptoms among junior high school students. They may need more external support and guidance to translate coping strategies into actual effects (35). Moreover, emotional regulation ability was not significantly associated with emotion-focused coping, which may reflect developmental variability in the relationship between emotional regulation and coping strategies during early adolescence (21). These findings may be consistent with age-related differences in coping processes and suggest that mediation patterns reported among older adolescents may not necessarily generalize to junior high school students (36).

From a theoretical perspective, the present findings expand the scope of application of the stress and coping model and emotional regulation theory in early adolescent populations. Most previous studies have supported the mediating role of coping styles among older adolescents or adults; in contrast, our study indicates that such mediation may not hold for junior high school students, thus emphasizing the importance of developmental sensitivity in mental health research. At the practical level, these results have clear and actionable implications for school-based mental health programs and family education. Consistent with the observed association between emotional regulation and adolescent mental health, emotional regulation ability may represent a relevant target for school- and family-based mental health programs, although intervention studies are needed to determine whether such training can reduce depressive symptoms (22, 29). Future school- and family-based programs could prioritize the evaluation of developmentally appropriate emotional regulation strategies, including emotional awareness, cognitive reappraisal, and constructive emotional expression (37). Although coping styles were not identified as significant mediators in this study, they remain important adaptive skills that are worth fostering; however, such training should be simplified, scenario-based, and tailored to the developmental level of early adolescents rather than adopting adult-oriented frameworks (38). For example, schools can implement 10–15-minute, classroom-based emotional regulation activities using age-appropriate scenarios—such as exam anxiety, peer conflicts, or parental pressure (39, 40). Teachers can guide students to practice concrete skills: recognizing emotional cues, applying simple cognitive reappraisal, doing slow breathing, or expressing feelings through structured talk or writing (38). For coping skills, brief role-plays help students identify adaptive responses, like seeking help during academic challenges, pausing before reacting, or breaking problems into manageable steps, rather than avoiding or lashing out (37). These short, context-relevant, and developmentally appropriate practices skip abstract adult theories, enabling junior high students to understand, practice, and apply emotional and coping skills in real life (38, 39).

Several limitations should be acknowledged. First, the cross-sectional design precludes causal or temporal inferences; longitudinal studies are needed to confirm dynamic relationships. However, this design aligns with standard preliminary psychosocial research in adolescents and is appropriate for examining concurrent associations and testing the proposed mediation model, supporting the robustness of our core finding: emotional regulation ability was negatively associated with depressive symptoms. Second, all data were self-reported, raising concerns about common method bias and social desirability. Yet the scales used are well-validated among Chinese junior high school students, with strong reliability and validity minimizing bias and preserving confidence in our key findings. Third, the sample was limited to junior high school students from Henan Province and was recruited using a convenience sampling approach, which may restrict the generalizability of the findings. Although Henan includes diverse urban-rural and socioeconomic backgrounds, the present results may not represent all Chinese junior high school students, particularly those from different geographic regions or educational environments with varying levels of academic pressure. Future studies involving larger and more diverse samples from multiple regions are needed to further validate these findings. Fourth, potential moderators (e.g., social support, family functioning, academic stress) were not included. Still, the model controlled for a comprehensive set of demographic covariates, adequately isolating the core relationships among emotional regulation, coping styles, and depressive symptoms without distorting observed patterns. Fifth, although participants were recruited within class clusters, the statistical models did not account for clustering because class-level identifiers were unavailable in the final analytic dataset. Ignoring within-class correlation may have resulted in underestimated standard errors and overly narrow confidence intervals, potentially overstating the statistical precision of some associations. Future studies should retain cluster identifiers and apply cluster-robust or multilevel analytical methods. Future studies should use longitudinal or experimental designs, incorporate multi-informant assessments, examine moderating factors, and recruit more diverse samples to verify and extend these findings.

This multi-school cross-sectional study identified a significant negative association between emotional regulation ability and depressive symptoms in early adolescence, while finding no significant mediating role for problem-focused or emotion-focused coping. These results may reflect developmental specificity, as emotional regulation ability showed a clear association with depressive symptoms, whereas neither coping dimension demonstrated a significant mediating effect at this developmental stage. Beyond the local sample, the findings have broad implications for global adolescent mental health promotion, especially in high-pressure educational settings. These findings provide a basis for further evaluating age-appropriate emotional regulation strategies in future school-based intervention studies and may inform mental health program development for early adolescents. Future intervention programs could consider emotional awareness, cognitive reappraisal, and contextually grounded skill practice tailored to the developmental characteristics of junior high school students. Future research should address key gaps: longitudinal designs to clarify causality and timing; intervention trials to test tailored emotional regulation programs; and cross-regional or cross-cultural studies to enhance generalizability. Examining moderators, including social support, family functioning, and academic stress will further refine the model and improve mental health support for early adolescents.

## Data Availability

The original contributions presented in the study are included in the article/supplementary material. Further inquiries can be directed to the corresponding author/s.
